# Immune Interaction Map of Human SARS-CoV-2 Target Genes: Implications for Therapeutic Avenues

**DOI:** 10.3389/fimmu.2021.597399

**Published:** 2021-03-16

**Authors:** Karthikeyan Subbarayan, Kamatchi Ulagappan, Claudia Wickenhauser, Michael Bachmann, Barbara Seliger

**Affiliations:** ^1^Institute of Medical Immunology, Martin Luther University Halle-Wittenberg, Halle, Germany; ^2^Institute of Pathology, Martin Luther University Halle-Wittenberg, Halle, Germany; ^3^Helmholtz-Zentrum Dresden-Rossendorf, Institute of Radiopharmaceutical Cancer Research, Dresden, Germany; ^4^German Cancer Consortium (DKTK), Partner Site Dresden, and German Cancer Research Center (DKFZ), Heidelberg, Germany; ^5^National Center for Tumor Diseases, University Hospital “Carl Gustav Carus,” Technische Universität (TU) Dresden, Dresden, Germany; ^6^Tumor Immunology, University Cancer Center “Carl Gustav Carus,” Technische Universität (TU) Dresden, Dresden, Germany; ^7^Cell therapy and Immunology, Fraunhofer Institute for Cell Therapy and Immunology, Leipzig, Germany

**Keywords:** SARS-CoV-2, therapeutics, DPP4, immune gene, bioinformatics

## Abstract

There exists increasing evidence that people with preceding medical conditions, such as diabetes and cancer, have a higher risk of infection with SARS-CoV-2 and are more vulnerable to severe disease. To get insights into the possible role of the immune system upon COVID-19 infection, 2811 genes of the gene ontology term “immune system process GO: 0002376” were selected for coexpression analysis of the human targets of SARS-CoV-2 (HT-SARS-CoV-2) ACE2, TMPRSS2, and FURIN in tissue samples from patients with cancer and diabetes mellitus. The network between HT-SARS-CoV-2 and immune system process genes was analyzed based on functional protein associations using STRING. In addition, STITCH was employed to determine druggable targets. DPP4 was the only immune system process gene, which was coexpressed with the three HT-SARS-CoV-2 genes, while eight other immune genes were at least coexpressed with two HT-SARS-CoV-2 genes. STRING analysis between immune and HT-SARS-CoV-2 genes plotted 19 associations of which there were eight common networking genes in mixed healthy (323) and pan-cancer (11003) tissues in addition to normal (87), cancer (90), and diabetic (128) pancreatic tissues. Using this approach, three commonly applicable druggable connections between HT-SARS-CoV-2 and immune system process genes were identified. These include positive associations of ACE2—DPP4 and TMPRSS2—SRC as well as a negative association of FURIN with ADAM17. Furthermore, 16 drugs were extracted from STITCH (score <0.8) with 32 target genes. Thus, an immunological network associated with HT-SARS-CoV-2 using bioinformatics tools was identified leading to novel therapeutic opportunities for COVID-19.

## Introduction

Coronaviruses (CoV) are a large family of viruses that cause illnesses ranging from a common cold to more severe diseases. A novel coronavirus (nCoV named SARS-CoV-2) belonging to this family was identified in 2019 in China and in March 2020 the WHO declared a worldwide pandemic of SARS-CoV-2 infection. As of February 13, 2021, more than 108 million cases have been confirmed, with more than 2.28 million deaths attributed to COVID-19.

Even though SARS-CoV and MERS-CoV have previously caused serious outbreaks, the intensity of these was much lower than the ongoing SARS-CoV-2 pandemic. Recent studies have demonstrated that SARS-CoV-2 utilizes the angiotensin-converting enzyme II (ACE-2) receptor (1, 2), MERS-CoV, the dipeptidyl peptidase-4 (DPP4/CD26) as an entry (3). The transmembrane serine protease 2 (TMPRSS2) and the proprotein convertase FURIN cleave and activate the viral glycoprotein spike (S), which facilitates virus-cell membrane fusion. SARS-CoV-2 requires both, the ACE2 receptor and TMPRSS2 for protein priming to enter the cell (4), while FURIN facilitates the active binding of SARS-CoV-2 through the ACE2 receptor (5), which is a risk factor for a more severe cause of COVID-19 (4, 6).

However, there exists limited information on why SARS-CoV-2 is more lethal and spreads faster than its two ancestors and how the immune system responds to SARS-CoV-2 infection. Recent publications have reported differences in the genome structure of the CoV family members (7) and immunological responses next to inflammation (5, 8). The viral entry has been shown to cause a cytokine storm, which may attack lung cells (9). This results in fluid filling the air sacks and alveolar cell apoptosis causing respiratory insufficiency, ultimately leading to death (10). Some strategies have been developed for the treatment of SARS-CoV-2 infection, such as potential vaccines and drugs targeting SARS-CoV-2 or host cell components, e.g., host cell receptors, necessary for viral replication. The complete knowledge of interactions between proteins in SARS-CoV-2 infected cells represents an essential milestone toward understanding the cellular mechanisms and functions of proteins including the genes involved in immune responses against virus infection (11). The gene ontology term (GO: 0002376) comprises genes involved in the development and/or function of the immune system that are necessary for the calibration of immune responses to potential internal or invasive threats.

The outbreak of SARS-CoV-2 is an ongoing global public health crisis. A study analyzing the impact of SARS-CoV-2 infection on patients with cancer demonstrated that the all-cause mortality within 30 days was high and a general risk factor for patients with active or prior cancer and confirmed SARS-CoV-2 infection (12). The demographic data from Italy showed that among 3,000 reported COVID-19 cases, 20% of the patients who died had a medical history of malignancies during the previous 5 years (13). A systematic review and meta-analysis of eight studies in predominantly Chinese populations (*n* = 46,248, searches run February 25, 2020) and six studies (*n* = 1,527) found diabetes with a frequency of 8% (95% CI 6–11%) and 9.7% (6.9–12.5%), respectively (14).

The demographic data relating to patients and their past medical history are crucial for the development of effective treatment opportunities. Therapeutic targets are urgently needed to manage COVID-19. One possibility is targeting the expression, the transcriptional regulation, or activity of host receptors and associated proteins known to play a critical role in the pathogenicity of CoV infections (4, 15–17). Indeed, studies using TMPRSS2 transgenic knockout mice have shown that the loss of TMPRSS2 reduces CoV replication in the lungs, and elicits a weaker proinflammatory response, and results in a milder lung pathology (15). In addition, SARS-CoV-2 entry into cells is also decreased upon functional inhibition of TMPRSS2 by the serine protease inhibitor camostat (4). Likewise, ACE2 antibodies or soluble recombinant ACE2 can attenuate viral entry and infection by SARS-CoV-2 (4, 16). Thus, a better understanding of the regulatory mechanisms that control expression levels of HT-SARS-CoV-2 might be key for the development of effective novel treatments for SARS-CoV-2 infection.

DPP4/CD26 is a ubiquitous membrane-bound aminopeptidase and has multiple physiological functions, such as the T cell receptor-mediated activation and proliferation of T cells (18) as well as the regulation of glucose homeostasis. Its importance has been highlighted by the approval of DPP4 inhibitors as an established glucose-lowering therapy in type 2 diabetes (19). Modeling the structure of the SARS-CoV-2 spike protein predicts an interaction of DPP4 in addition to ACE2 (20, 21). Interestingly, a correlation between DPP4 and ACE2 was found to suggest that both membrane proteins are relevant for virus entry (22). Indeed, DPP4 acted as a CoV co-receptor, suggesting a similar mechanism for the entry of SARS-CoV-2 (23). The coexpression of ACE2 and DPP4 as receptors of the spike glycoprotein postulates that different human CoVs target similar cell types across different human tissues and explains the presence of comparable clinical features in patients infected with different CoVs. Evaluation of SARS-CoV infections revealed that the virus was not only present in the tissues of the lung, liver, kidney, and intestine, but also of the pancreas indicating the pancreas as a potential CoV target (24, 25).

In this study, the coexpression of HT-SARS-CoV-2 and immune system process genes was evaluated followed by STRING analysis to determine the functional interactions between HT-SARS-CoV-2 and immune system genes. The STRING database network integrates direct and indirect/functional protein-protein interactions, such as stable physical associations, transient binding, substrate chaining, information relay, and others (26). Despite many limitations due to the low number of patients, the retrospective nature of evidence, and limited patient follow-up, these data provide early insights into how the management of patients with cancer and/or diabetes mellitus might be affected by the SARS-CoV-2 pandemic. This is an important issue, particularly since cancer and diabetes mellitus are risk factors for disease progression and such patients were shown to present more severe symptoms and unfavorable outcomes upon SARS-CoV-2 infection (27–29).

## Materials and Methods

### Samples and Patients

The R2 Genomics Platform is a web-based genomics analysis and visualization platform and allows biomedical researchers to integrate, analyze and visualize clinical and genomics data. The transcriptome data of healthy and cancer patients were sourced from The Cancer Genome Atlas (TCGA) dataset (http://cancergenome.nih.gov/) and microarray data of the NCBI Gene Expression Omnibus (NCBI GEO) (30). The datasets were available on R2 Genomics Analysis and Visualization Platform (http://r2.amc.nl) and ImmuCo (31). Human samples and immune cell, subpopulations were then analyzed and samples included: (i) Normal Various−353 (32); (ii) Mixed Cancer−11003 (TCGA; https://www.cancer.gov/tcga); (iii) Pancreatic Islets—Normal−87 (33); (iv) Pancreas—Mixed Tumor−90 (34); (v) Pancreatic Islets—Diabetes−128 (35); (vi) B cell−38; (vii) CD4+ T cell−551; (viii) CD8+ T cell−149; (ix) DC−406; (x) macrophage−362; (xi) monocyte−427; (xii) NK−128; and, (xiii) PBMC−1921.

### Gene Ontology Enrichment Analyses

The Gene Ontology source (GO; http://geneontology.org) provides structured, computable knowledge regarding the functions and products of genes. GO enrichment analyses included three categories, biological process (BP), molecular function (MF), and cellular components (CC). For our studies, the GO term “immune system process (GO: 0002376)” was selected, which comprises 2811 genes.

### Networking Analyses and Drug Screening

The protein-protein network was constructed using the STRING Database (String v10) (36) as a protein-protein interaction network, which also analyzed functional enrichments. STRING consisted of 24.6 million proteins with more than 2,000 million interactions. The coexpression scores in STRING v10 were computed by employing a revised and improved pipeline. Using high throughput microarray gene expression data from the NCBI GEO, we mined databases and literature as well as predictions based on genomic context analysis. The gene expression values are then correlated gene-by-gene (Pearson correlation) and the resulting values were calibrated against common members in KEGG pathway maps to compute STRING scores. Text mining score was computed by parsed search of statistically relevant co-occurrences of gene names in the scientific texts (SGD, OMIM, FlyBase, PubMed). The parameters included in the analysis for screening targets were a medium confidence score of 0.4 and a medium FDR stringency of 5%.

In the search for significant drug molecules and determine potential drug-associated mechanisms upon SARS-CoV-2 infections, we employed the Search Tool for Interactions of Chemicals (STITCH, stitch.embl.de), a chemical protein interaction database that contains 0.5 million compounds and 9.6 million proteins. STITCH (“search tool for interactions of chemicals”) integrates information about interactions from metabolic pathways, crystal structures, binding experiments, and drug-target relationships. The inferred information from phenotypic effects, text mining, and chemical structure similarity is used to predict relations between chemicals. STITCH further allowed exploration of the network of chemical relations, also in the context of associated binding proteins. Each proposed interaction can be traced back to the source of that data.

### Correlation Analysis

Gene correlation analyses were performed with the R2: Genomics Analysis and Visualization Platform and ImmuCo. Correlation statistics were reflected by Pearson correlation coefficients and Pearson's r is a measure of linear correlation between two sets of data. It is the covariance of two variables; it is essentially a normalized measurement of the covariance, such that the result always has a value between −1 and 1. A *p*-value < 0.05 was considered statistically significant.

### Survival Analysis

PrognoScan (http://www.prognoscan.org/) is a large collection of publicly available cancer microarray datasets with clinical annotation and a tool for assessing the biological relationship between gene expression and prognosis. In the PrognoScan database, the association of gene expression with the survival of patients were evaluated by the minimum *p*-value approach. Briefly, patients were first arranged by expression levels of a given gene. They were then divided into high- and low-expression groups, and the risk differences between any 2 groups were estimated by the log-rank test. Meta-survival analysis of pancreatic cancers was computed and derived from the GENT2 (http://gent2.appex.kr/gent2/).

### Statistical Analysis

Statistical computations and drawings of figures were performed with several packages (ggplot2 and circlize) in the statistical software environment R, version 3.3.2 (http://www.r-project.org).

## Results

### Identification of Protein Associations

The three human proteins ACE2, TMPRSS2, and FURIN used by SARS-CoV-2 to efficiently enter into human cells were studied for their functional protein associations using the STRING database. TMPRSS2 was networked as a center point associated with both ACE2 and FURIN, while ACE2 and FURIN showed no interactions between each other (Supplementary Figure 1). To study the immune system interactions of the human targets, the GO term immune system processes (GO: 0002376) in the Gene Ontology resource was analyzed.

**Figure 1:**
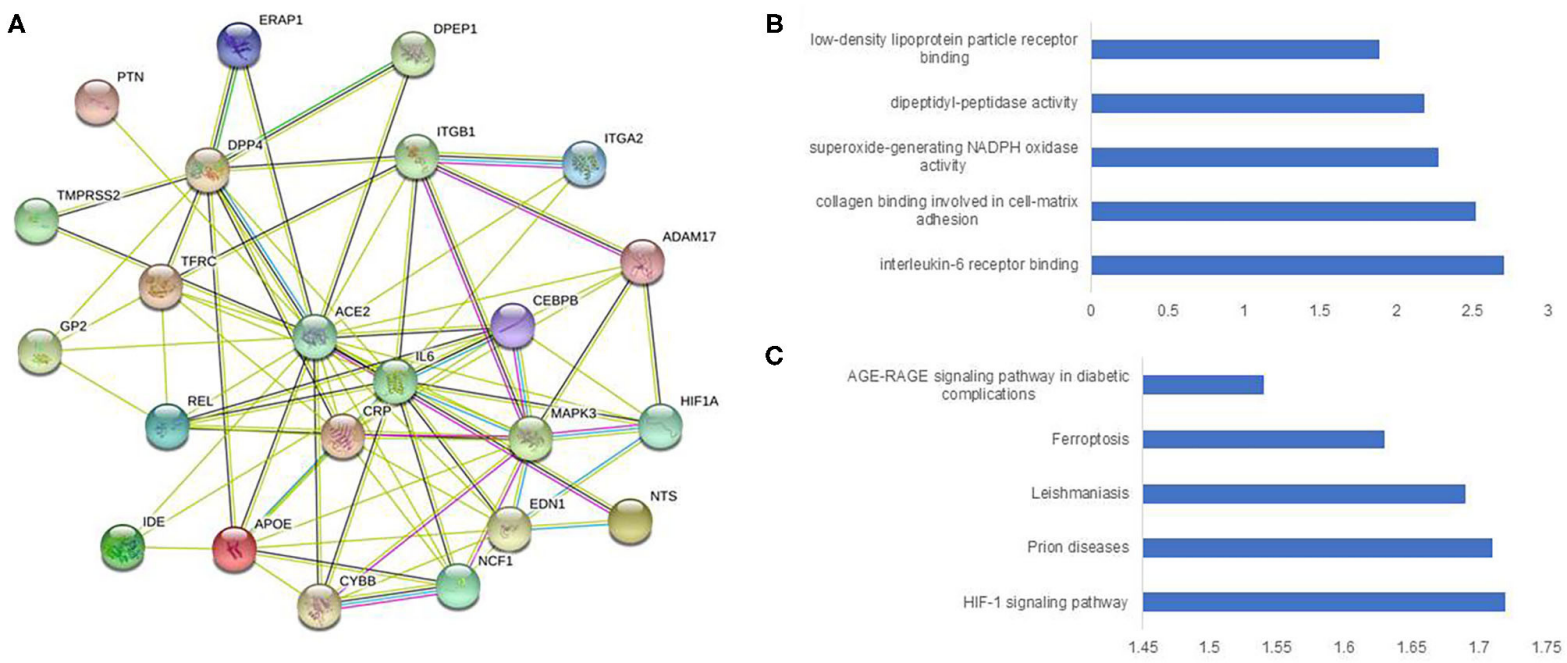
Construction and analysis of a protein-protein interaction network of ACE2 and immune genes. The protein-protein interaction network was visualized by STRING. The color saturation of the edges represents the confidence score of a functional association. **(A)** Intricate protein-protein interaction network (number of nodes 23, number of edges 80, average node degree 6.96, PPI enrichment *p*-value < 1.0e-16, average local clustering co-efficient 0.777). **(B)** Enrichment analysis by molecular function. **(C)** Enrichment analysis by KEGG.

In total, 2811 genes (Supplementary Table 1) were studied for functional protein association networks using STRING (string-db.org). This analysis produced 24,072 protein associations (Supplementary Table 2), among which, ACE2 shared 21 protein associations (Figure 1A) and protein-protein interaction (PPI) network analysis revealed 23 nodes, 80 edges, and an average node degree of 6.96. The PPI enrichment *p*-value was < 1.0e-16, whereas the average local clustering co-efficient was 0.777. The PPI of ACE2 enriched notably for IL-6 binding at molecular function (Figure 1B) and AGE-RAGE signaling pathway in diabetic complications at KEGG (Figure 1C). TMPRSS2 shared 17 protein associations (Supplementary Figure 2A) and the PPI displayed 18 nodes, 62 edges, and an average node degree of 6.89 with average local clustering co-efficient 0.797 (PPI enrichment *p*-value 5.35e-10). This network was the highest enriched for histone acetyltransferase binding at molecular function (Supplementary Figure 2B), and highest enriched for different cancer types at KEGG (Supplementary Figure 2C).

**Figure 2:**
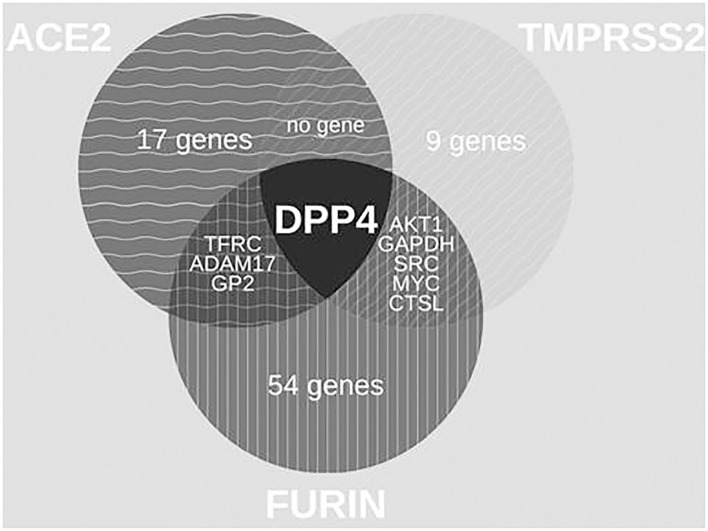
Venn diagrams of common protein associations between HT-SARS-CoV-2 and immune genes. The association between the HT-SARS-CV-2 ACE2, TMPRSS2, and FURIN and 2811 immune genes is demonstrated showing only one common immune molecule associated with all three HT-SARS-CoV-2.

With 64 proteins, FURIN indicated the highest associations (number of edges 464, average node degree 14.3, average local clustering coefficient 0.731, PPI enrichment *p*-value < 1.0e-16) (Supplementary Figure 3A). The PPI of FURIN was enriched at the molecular function for receptor activity and binding. KEGG was enriched at bladder cancer (Supplementary Figure 3B) and Notch signaling pathway (Supplementary Figure 3C). Among the protein associations within the three HT-SARS-CoV-2, DPP4 shared associations with ACE2, TMPRSS2, and FURIN (Figure 2), while ADAM17, glycoprotein 2 (GP2), transferrin receptor 1 (TFGC) were the common network elements with ACE2 and FURIN. CTSL, GAPDH, MYC, AKT1, and SRC were functionally associated with TMPRSS2 and FURIN (Table 1).

**Table 1:** Identification of immune process relevant genes associated with three HT-SARS-CoV-2.

**Gene A**	**Gene B**	**String**		
		**Coexpression**	**Text mining**	**Score**
**Associations with human targets of SARS-CoV-2**				
ACE2	TMPRSS2	0.099	0.39	0.427
TMPRSS2	FURIN	0	0.531	0.531
**Triple associations of DPP4**				
ACE2	DPP4	0.061	0.717	0.942
TMPRSS2	DPP4	0.062	0.396	0.409
FURIN	DPP4	0.061	0.424	0.439
**Double associations with ACE2 and FURIN**				
ACE2	ADAM17	0	0.706	0.707
FURIN	ADAM17	0.063	0.619	0.628
ACE2	GP2	0	0.571	0.571
FURIN	GP2	0	0.859	0.859
ACE2	TFRC	0	0.663	0.663
FURIN	TFRC	0.063	0.498	0.514
**Double associations with TMPRSS2 and FURIN**				
TMPRSS2	CTSL	0.061	0.469	0.48
FURIN	CTSL	0.062	0.49	0.514
TMPRSS2	MYC	0	0.518	0.518
FURIN	MYC	0.061	0.415	0.427
TMPRSS2	AKT1	0	0.472	0.476
FURIN	AKT1	0.106	0.429	0.479
TMPRSS2	SRC	0	0.4	0.405
FURIN	SRC	0.095	0.6	0.631
TMPRSS2	GAPDH	0	0.425	0.448
FURIN	GAPDH	0	0.414	0.414

*The association of HT-SARS-Cov-2 with immune relevant genes was determined by STRING analyses and represented as coexpression, text mining, and score*.

Although no genes were coexpressed at significant levels (*p* ≤ 0.05, Table 1), DPP4 was coexpressed with the three HT-SARS-CoV-2 analyzed with a *p*-value of 0.06. Likewise, FURIN with ADAM17 (*p* 0.063) and MYC (*p* 0.061) and CTSL with TMPRSS2 (*p* 0.061) and FURIN (*p* 0.062) shared coexpression patterns. The top text-mining score was obtained by FURIN/GP2 (0.859), followed by ACE2/DPP4 (0.717) and ACE2/ADAM17 (0.706). On the other hand, ACE2/DPP4 had the highest STRING score (0.942) followed by FURIN/GP2 (0.859) and ACE2/ADAM17 (0.707) (Table 1).

The STRING network analysis screened 102 of the HT-SARS-CoV-2association genes from the 2811 immune system process' genes.

### Determination of Genes Correlating With HT-SARS-CoV-2 in Cancer

The ACE2, TMPRSS2, and FURIN expression were correlated to every single gene in the five different datasets described in the Materials and Methods section above. The top ~1500 genes of the respective association, i.e., having a highly significant correlation (*p* < 0.01) in the corresponding dataset, are summarized in Supplementary Table 3. From the common STRING associations between the human target genes, 19 associations of eight commonly networking genes were plotted to the three HT-SARS-CoV-2 (Table 2) in mixed healthy (323 samples) and cancer (11003 samples) tissues.

**Table 2:** Association of HT-SARS-CoV-2 with immune process relevant genes in mixed cancers vs. mixed normal tissues.

**Gene A**	**Gene B**	**Mixed-normal**		**Mixed-cancers**	
		***R***	***P***	***R***	***P***
**Associations with human targets of SARS-CoV-2**					
ACE2	TMPRSS2	0.29	8.63E-07	0.164	1.42E-67
TMPRSS2	FURIN	0.376	2.78E-12	0.29	1.47E-211
**Triple associations of DPP4**					
ACE2	DPP4	0.308	1.66E-07	0.382	0.00E+00
TMPRSS2	DPP4	0.524	4.05E-24	0.382	0.00E+00
FURIN	DPP4	0.498	6.96E-20	0.246	1.82E-150
**Double associations with ACE2 and FURIN**					
ACE2	ADAM17	−0.187	4.22E-04	−0.044	3.76E-06
FURIN	ADAM17	−0.281	7.96E-07	−0.079	7.59E-17
ACE2	GP2	0.108	0.043	−0.045	2.64E-06
FURIN	GP2	0.006	0.905	0.098	8.87E-25
ACE2	TFRC	0.085	0.11	0.085	4.23E-19
FURIN	TFRC	0.057	0.286	0.05	1.61E-07
**Double associations with TMPRSS2 and FURIN**					
TMPRSS2	CTSL	0.111	0.038	0.129	3.93E-42
FURIN	CTSL	0.154	3.83E-03	0.175	1.44E-76
TMPRSS2	MYC	0.274	1.74E-07	0.022	2.20E-02
FURIN	MYC	0.149	5.04E-03	0.012	0.199
TMPRSS2	AKT1	0.274	1.71E-07	0.137	3.43E-47
FURIN	AKT1	0.193	2.67E-04	0.21	2.30E-109
TMPRSS2	SRC	0.288	3.75E-08	0.309	1.86E-240
FURIN	SRC	0.161	2.42E-03	0.084	1.51E-18
TMPRSS2	GAPDH	−0.308	3.28E-09	−0.314	7.39E-250
FURIN	GAPDH	−0.198	1.87E-04	0.023	1.80E-02

*Correlation of HT-SARS-Cov2 with immune process relevant genes was performed by RZ2 genomics using mixed healthy and mixed cancer data sets and presented as correlation values (R) including the statistical significance (P)*.

**Figure 3:**
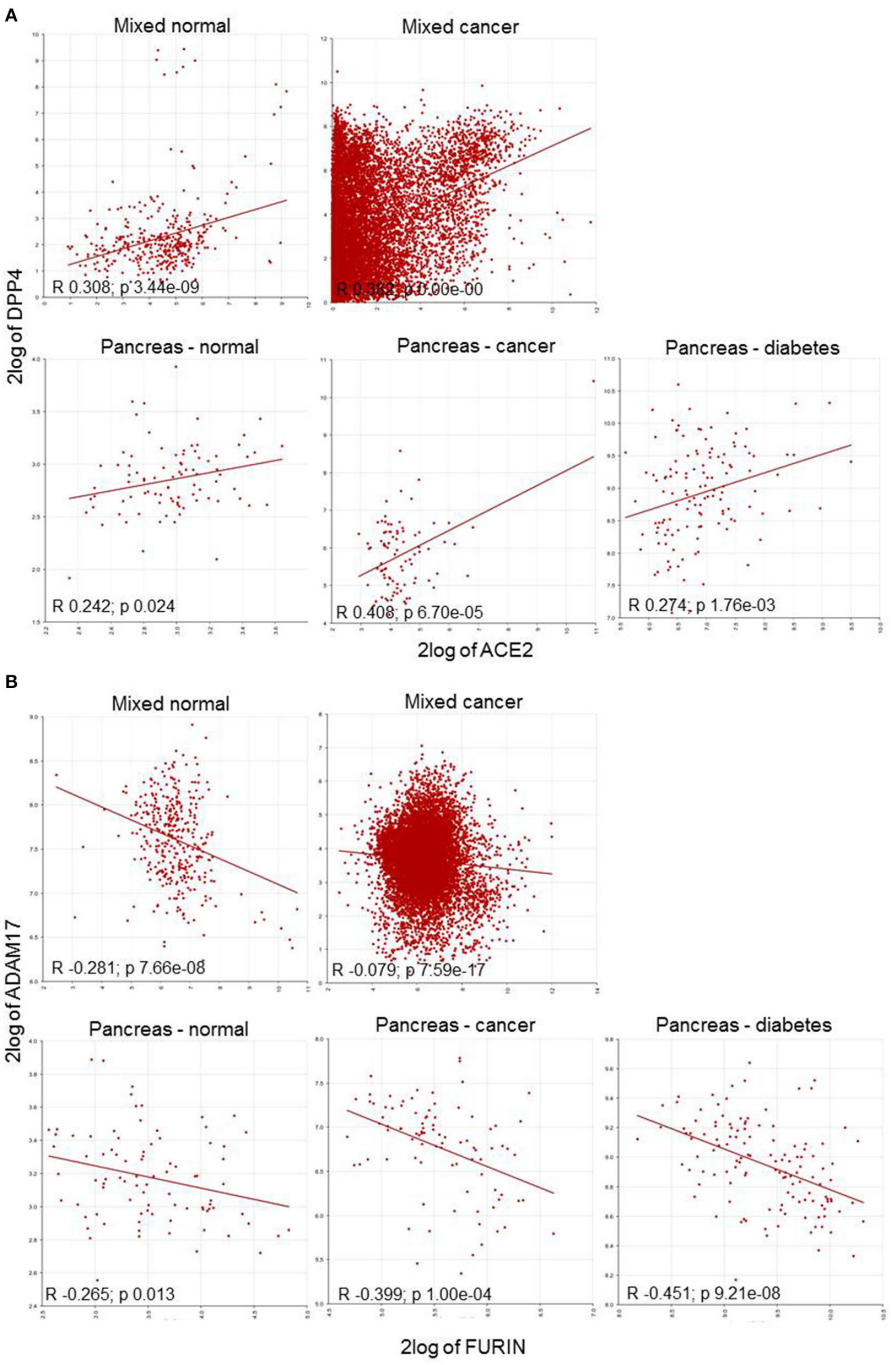
Correlation plot of HT-SARS-CoV-2 genes in mixed normal and cancers, and pancreatic normal, cancer, and diabetic tissue. **(A)** Positive correlations of ACE2/DPP4. **(B)** Negative correlations of FURIN/ADAM17. 2log: logarithmic values with base of 2.

This collected data enabled classification into four distinct categories: (i) associations between human target genes; (ii) triple association of DPP4; and, (iii) double associations of FURIN with either ACE2; or (iv) with TMPRSS2, respectively. The top segment of Table 2 represents the positive correlations involving TMPRSS2 in both healthy and tumor datasets. The central association marker, DPP4 positively correlated with ACE2 (normal: *R* 0.308; cancers: 0.382), TMPRSS2 (normal: *R* 0.524; cancers: 0.382) and FURIN (normal: *R* 0.498; cancers: *R* 0.246) in both datasets (Figure 3). ADAM17 exhibited negative correlations with ACE2 (normal: *R* −0.187; cancers: *R* −0.044) and FURIN (normal: *R* −0.281; cancers: *R* −0.079). GP2 and TFRC showed differential association patterns with ACE2 and FURIN. The common TMPRSS2 and FURIN associated genes CTSL, MYC, AKT1, and SRC displayed positive correlations with their corresponding subjects except for the FURIN and MYC correlation in cancers (*R* 0.012; *p* 0.199). TMPRSS2 negatively correlated with GAPDH (Table 2). Normal and cancer samples mostly followed the same pattern. However, ACE2/GP2 (*R* 0.108 in normal and *R* −0.045 in cancer) and FURIN/GAPDH (*R* −0.198 in normal and *R* 0.023) present differential correlations between normal and cancer samples.

To explore gene association patterns in the wide range of datasets, ACE2, TMPRSS2, and FURIN were correlated to DPP4, SRC, and ADAM17 expression regardless of the tissue and the disease. The analysis demonstrated that ACE2 and TMPRSS2 displayed a positive correlation, whereas FURIN showed a negative correlation with their counterparts (Table 3 and Figure 3).

**Table 3:** Associations of HT-SARS-CoV-2 with immune process relevant genes in normal pancreas, pancreatic cancer, and diabetic pancreatic tissues.

**Pancreas**		**Normal**		**Cancer**		**Diabetes**	
**Gene A**	**Gene B**	***R***	***P***	***R***	***P***	***R***	***P***
**Associations with human targets of SARS-CoV-2**							
ACE2	TMPRSS2	0.22	4.10E-02	0.147	1.66E-01	0.104	0.245
TMPRSS2	FURIN	−0.008	9.44E-01	0.306	3.39E-03	0.41	1.51E-06
**Triple associations of DPP4**							
ACE2	DPP4	0.242	2.40E-02	0.408	6.70E-05	0.274	1.76E-03
TMPRSS2	DPP4	0.376	3.28E-04	−0.391	1.37E-04	−0.244	5.52E-03
FURIN	DPP4	0.019	8.63E-01	−0.485	1.24E-06	0.077	3.88E-01
**Double associations with ACE2 and FURIN**							
ACE2	ADAM17	0.262	1.40E-02	−0.076	4.79E-01	0.076	3.91E-01
FURIN	ADAM17	−0.265	1.30E-02	−0.399	1.00E-04	−0.451	9.21E-08
ACE2	GP2	0.307	0.00384	0.039	0.718	0.099	2.65E-01
FURIN	GP2	−0.415	6.31E-05	0.715	2.46E-15	−0.098	2.71E-01
ACE2	TFRC	0.313	0.00312	−0.02	0.854	0.052	5.62E-01
FURIN	TFRC	−0.152	0.159	−0.425	2.97E-05	−0.737	3.35E-23
**Double associations with TMPRSS2 and FURIN**							
TMPRSS2	CTSL	0.419	5.44E-05	−0.458	5.78E-06	−0.086	3.35E-01
FURIN	CTSL	−0.094	3.88E-01	−0.21	4.70E-02	−0.114	2.01E-01
TMPRSS2	MYC	0.048	6.61E-01	−0.002	9.85E-01	0.308	4.12E-04
FURIN	MYC	0.358	6.60E-04	0.234	2.60E-02	−0.042	6.36E-01
TMPRSS2	AKT1	0.233	3.00E-02	−0.101	3.46E-01	0.236	7.28E-03
FURIN	AKT1	−0.093	3.91E-01	0.335	1.25E-03	0.882	6.15E-43
TMPRSS2	SRC	0.437	2.27E-05	0.378	2.38E-04	0.567	3.01E-12
FURIN	SRC	−0.05	6.46E-01	0.102	3.40E-01	0.487	5.74E-09
TMPRSS2	GAPDH	−0.229	3.30E-02	−0.224	3.30E-02	−0.082	3.60E-01
FURIN	GAPDH	0.565	1.19E-08	−0.32	2.09E-03	0.347	5.93E-05

*Correlation of HT-SARS-CoV-2 with immune process relevant genes was performed by R2 genomics using data sets obtained from normal pancreatic, pancreatic cancer, and diabetic pancreatic tissues. Data are presented as correlation values (R), including the statistical significance (P)*.

### Correlation Pattern of HT-SARS-CoV-2 in Pancreatic Tissues

ACE2 expression in the pancreas was reported to cause pancreatic damage after SARS-CoV-2 infection (37). Interestingly, ACE2 and DPP4 were connected irrespective of the pancreatic nature i.e., healthy, cancer and diabetes mellitus. The healthy pancreatic tissues exhibited distinct associations when compared to pancreatic cancer tissues or tissue samples from patients suffering from diabetes. DPP4 was differentially expressed with TMPRSS2 and FURIN, while TMPRSS2 was highly expressed in healthy and only scanty expressed in the diseased pancreatic tissue. The HT-SARS-CoV-2 ACE2 and TMPRSS2 were positively associated with healthy pancreatic tissue, while a non-significant positive association was noted in the context of cancer and diabetes mellitus. A positive correlation between TMPRSS2 and FURIN was found in the diseased, but not in the healthy pancreas (Table 3).

FURIN expression was not correlated with DPP4 except for pancreatic cancer, where a negative correlation was found. The FURIN/ADAM17 axis remained negatively associated with pancreatic tissues. Interestingly, TMPRSS2 positively correlated with SRC in the pancreatic tissues in mixed healthy and cancer datasets. Other double associated genes lacked any specific correlation pattern. FURIN and MYC were positively associated with healthy tissues and pancreatic cancer. However, FURIN positively acted with MYC in pancreatic cancer, but not in the mixed cancer dataset. AKT1 with TMPRSS2 and FURIN were positively correlated in all contexts except for pancreatic cancer and healthy pancreas, respectively (Table 3). Interestingly, many networking genes were not correlated in pancreatic tissues. These included ACE2/ADAM17, FURIN/GAPDH, ACE2/FURIN, ACE2/GP2, and ACE2/TFRC, respectively.

### Correlation Pattern of HT-SARS-CoV-2 in Peripheral Blood Mononuclear Cells and Immune Cells

The impact of HT-SARS-CoV-2 on immune responses were analyzed in PBMC and different immune cell subpopulations. In agreement with the mixed normal and tumor datasets, PBMC displayed positive correlations between ACE2/TMPRSS2, TMPRSS2/FURIN, ACE2/TFRC, FURIN/TFRC, TMPRSS2/CTSL, FURIN/CTSL, TMPRSS2/MYC, and FURIN/AKT1 and a negative association between ACE2/ADAM17. Interestingly, FURIN/ADAM17 and TMPRSS2/GAPDH showed a positive correlation (Table 4 and Supplementary Table 4).

**Table 4:** Associations of HT-SARS-CoV-2 with immune process relevant genes in PBMC, B cells, and T cells.

**Immune cells**		**PBMC**		**B cell**		**CD4+** **T cell**		**CD8+** **T cell**	
**Gene A**	**Gene B**	***R***	***P***	***R***	***P***	***R***	***P***	***R***	***P***
ACE2	TMPRSS2	0.194749	0.00E+00	0.563	2.31E-04	0.207922	1.00E-06	0.397018	1.00E-06
TMPRSS2	FURIN	0.052281	0.021933	0.584	0.000117	0.015903	7.10E-01	−0.031942	6.99E-01
ACE2	DPP4	0.040884	7.32E-02	0.316	5.30E-02	0.072992	8.69E-02	−0.141403	8.54E-02
TMPRSS2	DPP4	0.040639	0.074956	0.352	3.00E-02	−0.015323	7.20E-01	−0.151717	6.47E-02
FURIN	DPP4	−0.035395	1.21E-01	0.301	6.60E-02	0.470333	0.00E+00	0.377543	2.00E-06
ACE2	ADAM17	−0.214355	0.00E+00	−0.488	1.86E-03	−0.00236	9.56E-01	−0.172781	3.51E-02
FURIN	ADAM17	0.282705	0.00E+00	−0.488	1.90E-03	0.088845	0.037081	0.271443	8.12E-04
ACE2	GP2	0.231479	0.00E+00	0.693	0.00000145	0.362058	0	0.329296	4.10E-05
FURIN	GP2	0.01108	6.27E-01	0.519	0.000848	−0.073753	0.08369	0.07534	3.61E-01
ACE2	TFRC	0.188254	0.00E+00	−0.393	0.015	0.028397	0.505932	0.094183	2.53E-01
FURIN	TFRC	0.158378	0.00E+00	−0.512	0.00102	0.395723	0	0.331861	3.60E-05
TMPRSS2	CTSL	0.096595	2.20E-05	−0.047	7.81E-01	0.033176	4.37E-01	−0.067532	4.13E-01
FURIN	CTSL	0.134294	0.00E+00	0.138	4.10E-01	−0.119595	4.94E-03	0.252604	1.88E-03
TMPRSS2	MYC	0.076205	8.30E-04	−0.614	4.12E-05	0.09934	1.97E-02	−0.023417	7.77E-01
FURIN	MYC	−0.216085	0.00E+00	−0.484	2.08E-03	0.272609	0.00E+00	0.054391	5.10E-01
TMPRSS2	AKT1	0.04259	6.20E-02	0.305	6.30E-02	0.062252	1.44E-01	0.082334	3.18E-01
FURIN	AKT1	0.282536	0.00E+00	0.256	1.20E-01	0.412729	0.00E+00	0.206321	1.16E-02
TMPRSS2	SRC	0.118741	0.00E+00	0.642	1.37E-05	0.012309	7.73E-01	0.161731	4.88E-02
FURIN	SRC	−0.005434	8.12E-01	0.578	1.44E-04	0.114058	7.36E-03	−0.036473	6.59E-01
TMPRSS2	GAPDH	0.166781	0.00E+00	No data	0.111217	8.98E-03	0.096452	2.42E-01	
FURIN	GAPDH	−0.104979	0.000004	No data	0.392612	0	0.05329	0.518622	

*Correlation of HT-SARS-CoV-2 with immune process relevant genes was performed by ImmuCo using data sets obtained from PBMC, B cells, and T cells. Data are presented as correlation values (R), including the statistical significance (P)*.

ACE2 and TMPRSS2 were positively correlated in immune cells like B cells, T cells, dendritic cells (DC), macrophages, monocytes, and NK cells. In contrast, TMPRSS2 and FURIN exhibited positive associations in B cells and monocytes, while other immune cell subpopulations lacked any correlations. Except for B cells, immune cells displayed no correlations with the central association markers, DPP4/ACE2 or TMPRSS2. FURIN and DPP4 revealed positive correlations in CD4^+^ and CD8^+^ T cells and negatively correlated in DC and monocytes. FURIN and ADAM17 showed a general significant association with a positive association in B cells, T cells, monocytes, and NK cells, but negative correlations in DC and macrophages (Table 4 and Supplementary Table 4). Irrespective of any immune cell type, ACE2 and GP2 were significantly positively correlated to each other. It is noteworthy that many networking genes were not associated with the immune cells under study.

### Identification of Drug Candidates for HT-SARS-CoV-2 and Co-expressed Genes

**Figure 4:**
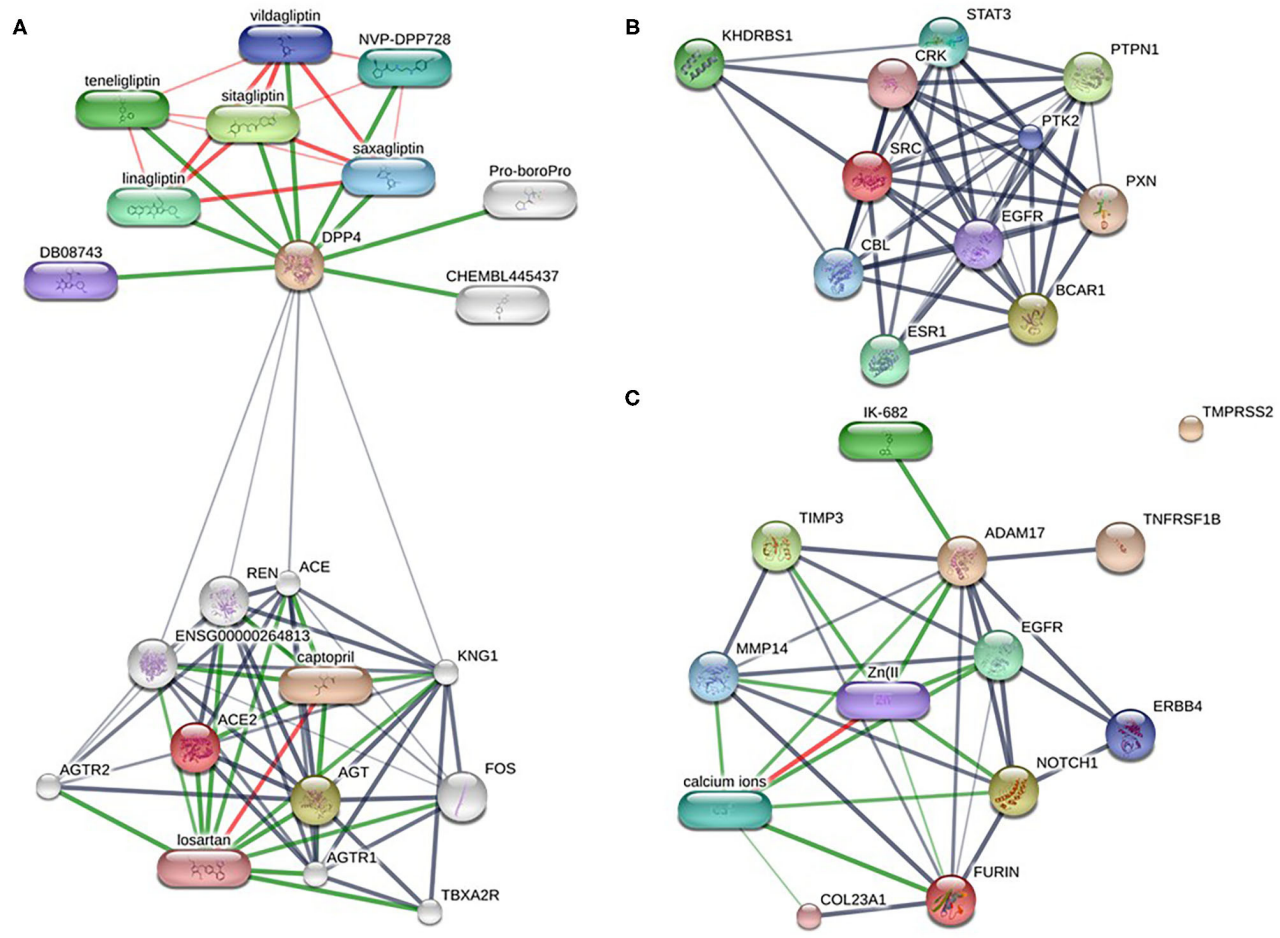
Drug-target networks of HT-SARS-CoV-2 using the STITCH database. The druggable targets of the association of HT-SARS-CoV-2 with immune process genes were curated from the STITCH database. Pill-shaped and spheres nodes represent the compounds and proteins, respectively. **(A)** ACE2/DPP4. **(B)** TMPRSS2/SRC. **(C)** FURIN/ADAM17.

Altogether ~0.5 million compounds were studied on the STITCH database. Among these compounds, 16 target drugs were found according to the screening conditions (Table 5). The active components involved 32 drug-associated genes (Supplementary Table 4), which were acquired by STITCH analysis. As shown in Figure 4, ACE2/DPP4 (Figure 4A) and FURIN/ADAM17 (Figure 4C) were predicted to have drug connections, whereas TMPRSS2/SRC (Figure 4B) had no predicted drug connection. Several inhibitors of the angiotensin receptor (ACE) and DPP4, in particular gliptin members, such as sitagliptin, teneligliptin, linagliptin, vildagliptin, and saxagliptin, were identified. For FURIN and ADAM17, a therapeutic involvement *via* calcium ions and Zn (II) and for ADAM17, an involvement *via* IK-682 were identified (Table 5). Among these three interactions, two druggable connections were suggested for a putative therapeutic option.

**Table 5:** Known drug candidates targeting HT-SARS-CoV-2 and associated genes.

**gene**	**Drug**	**Exp. determined** ** interaction**	**Database** ** annotated**	**Automated** ** text mining**	**Combined score**
ACE2	Losartan	0	0.900	0.907	0.990
ACE2	Captopril	0.790	0.900	0.515	0.989
DPP4	Sitagliptin	0.900	0.800	0.968	0.999
DPP4	Teneligliptin	0.972	0.800	0.865	0.999
DPP4	Linagliptin	0.937	0.800	0.920	0.998
DPP4	NVP-DPP728	0.915	0.800	0.893	0.998
DPP4	Vildagliptin	0.797	0.800	0.932	0.997
DPP4	saxagliptin	0.807	0.800	0.933	0.997
DPP4	DB08743	0.957	0.800	0	0.991
DPP4	CHEMBL445437	0.989	0	0	0.989
DPP4	Pro-boroPro	0.912	0.800	0.395	0.988
FURIN	calcium ions	0.886	0.900	0.133	0.989
URIN	Zn(II	0.498	0	0.120	0.545
ADAM17	IK-682	0.978	0.800	0.169	0.996
ADAM17	Zn(II	0.800	0.900	0.139	0.981
ADAM17	calcium ions	0.800	0	0	0.800

*Drug candidates of HT-SARS-CoV-2, and immune process relevant genes were determined by STRING analysis and experimental interaction, database annotation, automatic text mining presented. Besides, the data were summarized in a combined score*.

### Survival Analysis on HT-SARS-CoV-2 and Druggable Targets

HT-SARS-CoV-2 and their drug connections were tested for their association with overall survival (OS) rates in 11 different cancer types (Supplementary Table 6). Although many cancer types did not show a significant survival ratio, ACE2 was significant in brain cancer (HR 0.44; *p* 0.003), breast cancer (HR 1.23; *p* 0.002), lung cancer (HR 0.7; *p* 0.009), ovarian cancer (HR 0.63; *p* 0.048) and renal cell carcinoma (HR 0.17; *p* 0.021). ADAM17 showed high hazard ratios in bladder (HR 4.65; *p* 0.04), brain (HR 3.65; *p* 0.0004) and colorectal (HR 2.0; *p* 0.024) cancers. The meta-survival analysis on pancreatic cancer datasets only showed significance in ADAM17 (HR 1.21; *p* 0.08) (Supplementary Figure 4). OS did not follow any relations with the correlation and networking of HT-SARS-CoV-2.

## Discussion

In this comprehensive study, the critical immune-related associations with the HT-SARS-CoV-2 ACE2, TMPRSS2, and FURIN were identified using bioinformatics analyses. SARS-CoV-2 requires the proteins ACE2, TMPRSS2, and FURIN for cell entry (4), which are the same proteins used by SARS-CoV (38), the cause of the 2002-2004 SARS epidemic. The SARS-CoV-2 viral genome encodes for 29 different proteins. A recent study by Gordon and et al. expressed and purified 26 of these proteins and analyzed them for protein-protein interactions (39). Using this approach, 332 high confident protein-protein interactions between SARS-CoV-2 and human proteins and 66 druggable human proteins were identified. Interestingly, two sets of pharmacological drugs had antiviral activities. These included inhibitors of mRNA translation of two sigma receptors.

Several drugs/drug classes were identified. Most of them are already used clinically for other indications. These include recombinant soluble ACE2, indirect ACE2 modulators (angiotensin receptor blockers, calmodulin antagonists, selective estrogen receptor modifiers), TMPRSS2 inhibitors (camostat mesylate, nafamostat mesylate, antiandrogens, inhaled corticosteroids), and ADAM-17 enhancers (5-fluorouracil).

FURIN and ADAM17 are both intertwined with Notch, an evolutionarily conserved communication system between adjacent cells, and thus targeting of Notch might represent an alternative approach to inhibit FURIN and upregulate ADAM17 (40). In this context, it is noteworthy that ADAM17 is involved in the shedding of ACE2 (41). Such hypothesis-driven studies based on the knowledge of the molecular details of virus-cell interaction are still crucial for the identification of therapeutic targets to treat COVID-19.

SARS-CoV-2 infection of pancreatic endocrine cells *via* ACE2 appears an unlikely central pathogenic feature of SARS-CoV-2 as it relates to diabetes (42). However, we found that ACE2 was highly expressed in pancreas islets. This could be an explanation for the finding that SARS-CoV-2 infections can cause damage of the islets and subsequent acute diabetes (25, 43). In our studies, we found that ACE2 and DPP4 were positively connected irrespective of the pancreatic nature, i.e., normal, cancer and diabetes. A positive correlation of ACE2 and DPP4 with TMPRSS2 was lacking in pancreatic cancer and diabetes.

Studies have shown a strong correlation between the severity of COVID-19 and concentrations of different cytokines, such as IL2, IL7, IL10, GCSF, MCP1, and TNF-alpha (44). Notch and IL-6 cooperate to activate the immune system and perpetuate the cytokine storm. In macrophages, Delta Like Canonical Notch Ligand 4 (Dll4)/Notch signaling promotes the production of inflammatory cytokines, including IL-6 (40). IL-6, in turn, increases the expression of the Notch ligands (Dll1 and DII4), thus amplifying the Notch signaling, thereby establishing a feedback loop that promotes the further production of IL-6. In T cells, Notch signaling triggered by Dll1/Dll4 ligands promotes inflammatory Th1/Th17 cytokines, while Jagged1 ligands dampen the IL-6-induced Th17 activation (40).

Currently, there are few reports on the specific mechanisms of interaction networks and associations of TMPRSS2, ACE2, and FURIN with their targets. Using bioinformatics tools, we here show that ACE2 may participate in Notch signaling to exert immune effects. However, the relationship between these molecules has to be analyzed and further experimentally validated concerning the possible targeting of ACE2/DPP4 and FURIN/ADAM17-related factors in diseased COVID-19 patients. The delicate balance between ACE2, ADAM17, and TMPRSS2 interactions could be decisive for the clinical outcome of COVID-19 (45).

Angiotensin receptor blockers (ARBs) similar to angiotensin-converting enzyme (ACE) inhibitors, act by preventing the formation of angiotensin II rather than by blocking the binding of angiotensin II to muscle cells on blood vessels. The therapeutic indication for ARBs prescription comprises control of high blood pressure, treating heart failure, and preventing kidney failure, especially in patients suffering from diabetes mellitus. Since ACE inhibitors are associated with cough, accumulation of bradykinin, and angioedema, ARBs, like losartan and captopril, might be a more favorable therapeutic option by blocking the binding and attachment of the SARS-CoV-2 receptor binding domain to ACE2-expressing cells, thereby inhibiting the infection of host cells (46). Ferrario and colleagues reported that the administration of either ACE inhibitors or ARBs increased the levels of ACE2 mRNA in Lewis rats, compared to rats receiving placebo (47). In particular, the cardiac levels of ACE2 mRNA expression increased by 4.7-fold or 2.8-fold in rats treated with losartan, which was accompanied by increased ACE2 activity. Treatment with the ACE inhibitor captopril can significantly increase ACE2 protein expression in rats with acute lung injury (48). Furthermore, Mourad and Levy suggested aliskiren treatment as an option in the context of SARS-CoV-2 infection, since it can reduce the expression of ACE2 (49).

Early trials in the 1990s showed that DPP4 inhibitors improve glycaemia in animals. Subsequent clinical studies during the 2000s showed a glucose-lowering authority of DPP4 inhibitors in humans with type 2 diabetes as monotherapy or in combination with other therapies, i.e., metformin, sulfonylureas, tiazolidinediones, or exogenous insulin. Five DPP-4 inhibitors (sitagliptin, vildagliptin, alogliptin, saxagliptin, and linagliptin) were approved by regulatory authorities for the treatment of type 2 diabetes and entered the market between 2006 and 2013 (50).

The inhibition of DPP4 led to the suppression of breast cancer tumor growth (51) and displayed a positive trend in OS of colorectal cancer (52) and prostate cancer (53). Analysis of colorectal cancer and lung cancer patients showed a similar trend toward beneficial effects associated with DPP4 inhibition (54); however, further studies are required on the clinical impact of DPP4 inhibition on tumor patients. Based on our results it is suggested that the modulation of DPP4 might interfere with the infection and/or progression of COVID-19 and therefore might represent a therapeutic strategy. Since DPP4 inhibitors have been shown to modulate inflammation and exert anti-fibrotic activity, they might have the potential to affect the hyperinflammatory state associated with severe COVID-19 (55).

## Conclusions

COVID-19 is a devastating novel disease resulting in thousands of deaths worldwide. It is essential to find causal therapies for those patients with a severe course, among them patients with comorbidities, as quickly as possible. The results of the present study suggest novel drug candidates for COVID-19 and underscore that new treatment options can be postulated by using different bioinformatics tools, which now have to be experimentally proven. Screening based on drug targets and their mechanisms is still key to developing new drugs. The present study provides comprehensive bioinformatics information on genes that are potential targets and candidate drugs for SARS-CoV-2. This information may help us to better understand the relevant molecular targeting mechanisms of this disease. However, further explorations are required to evaluate the biological functions of these biomarkers and drugs in the pathogenesis of the disease.

## Data Availability Statement

The original contributions generated in the study are included in the article/Supplementary Material, further inquiries can be directed to the corresponding author.

## Author Contributions

KS and BS designed the study and wrote the article. KU and KS performed the data analyses. MB and CW discussed the data and revised the manuscript. All authors contributed to the article and approved the submitted version.

## Conflict of Interest

The authors declare that the research was conducted in the absence of any commercial or financial relationships that could be construed as a potential conflict of interest.

## Abbreviations

ACE-2: angiotensin converting enzyme II
ADAM17: ADAM metallopeptidase domain 17
ARB: angiotensin receptor blockers
BP: biological process
CC: cellular component
CoV: coronavirus
COVID-19: coronavirus disease 2019
DII: Delta-Like Ligand
DPP4: dipeptidyl peptidase-4
GO: gene ontology
GP2: glycoprotein 2
HT-SARS-CoV-2: human targets of SARS-CoV-2
MF: molecular function
nCoV: novel coronavirus
OS: overall survival
PBMC: peripheral blood mononuclear cell
PPI: protein-protein interaction
SARS-CoV-2: severe acute respiratory syndrome coronavirus type 2
TFRC: transferrin receptor 1
TMPRSS2: transmembrane serine protease 2.
