# Supplementary material for: Immune Interaction Map of Human SARS-CoV-2 Target Genes: Implications for Therapeutic Avenues
Source: Front Immunol. 2021 Mar 16;12:597399. doi: 10.3389/fimmu.2021.597399 (PMC8007772; doi:10.3389/fimmu.2021.597399)

Supplementary Figure 4:

Meta-survival analysis on pancreatic cancers

ACE2

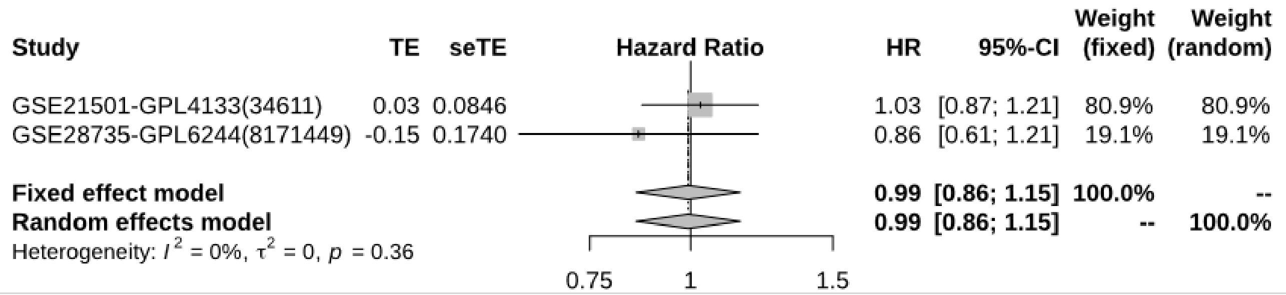

TMPRSS2

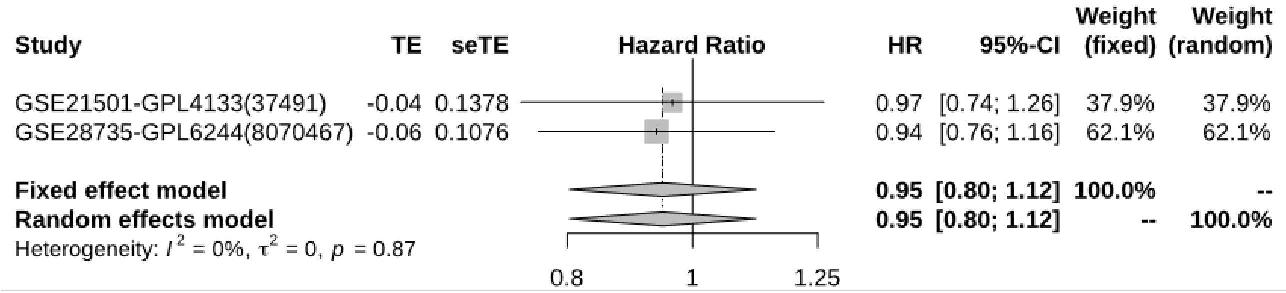

FURIN

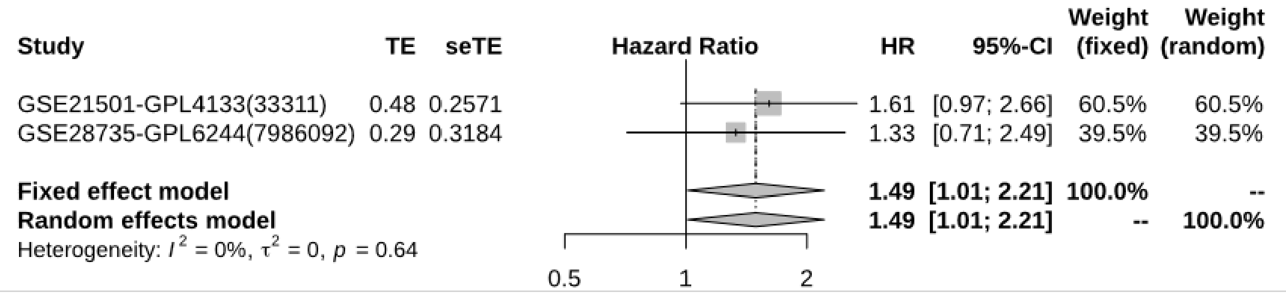

DPP4

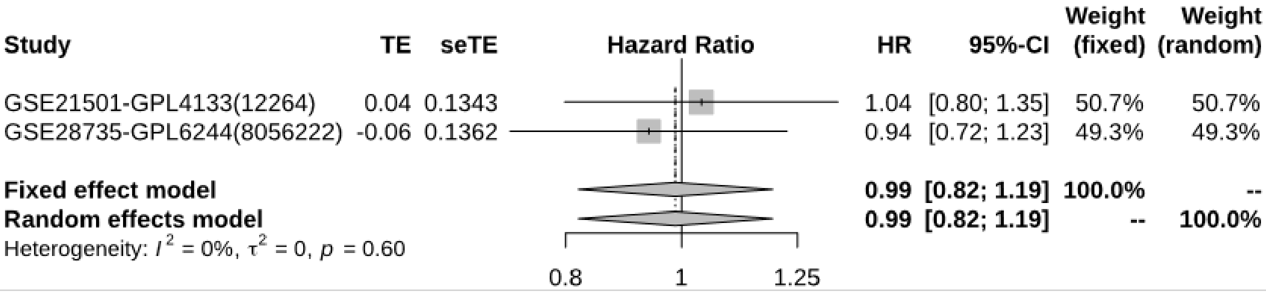

SRC

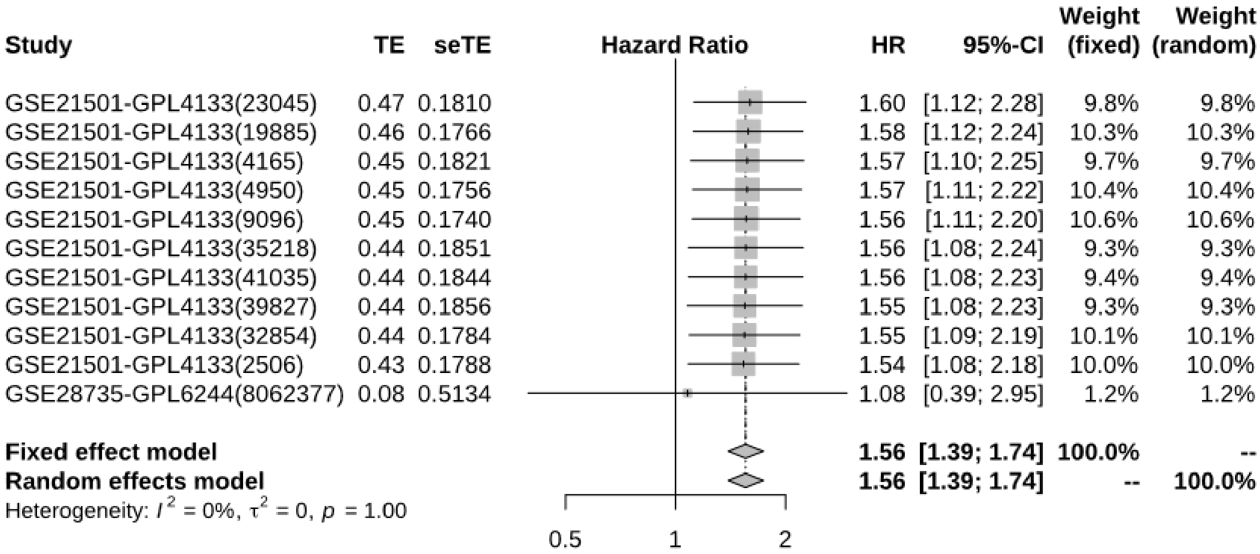

ADAM17

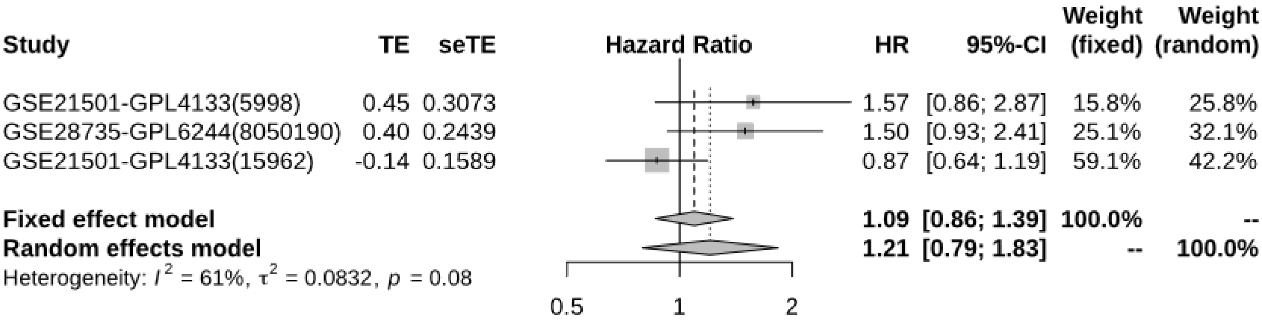

Supplement: Supplementary file 1 [file Data_Sheet_1.ZIP › Supplementary Figure 4.pdf]
