# Supplementary figures and images for: Immune Interaction Map of Human SARS-CoV-2 Target Genes: Implications for Therapeutic Avenues
Source: Front Immunol. 2021 Mar 16;12:597399. doi: 10.3389/fimmu.2021.597399 (PMC8007772; doi:10.3389/fimmu.2021.597399)

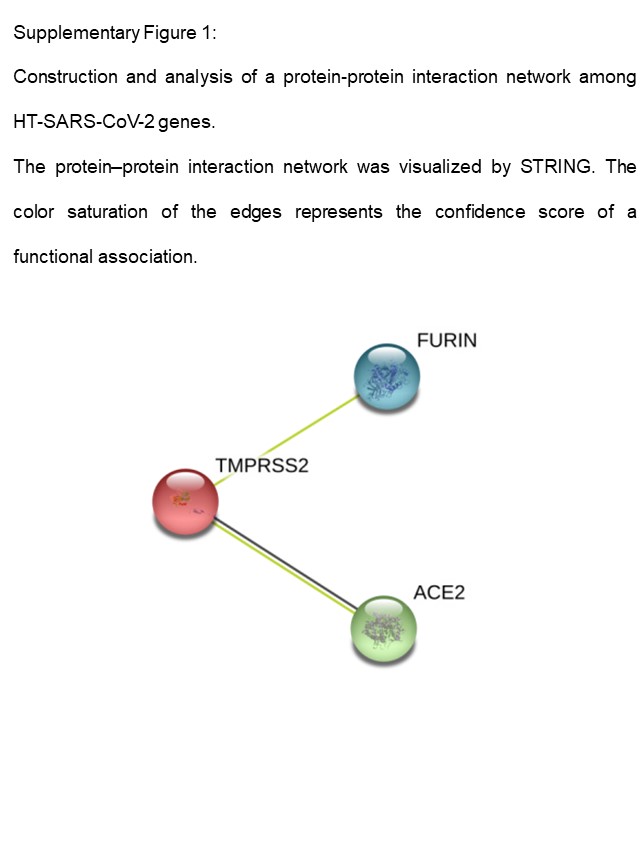

Supplement: Supplementary file 1 [file Data_Sheet_1.ZIP › Supplementary Figure 1.jpg]

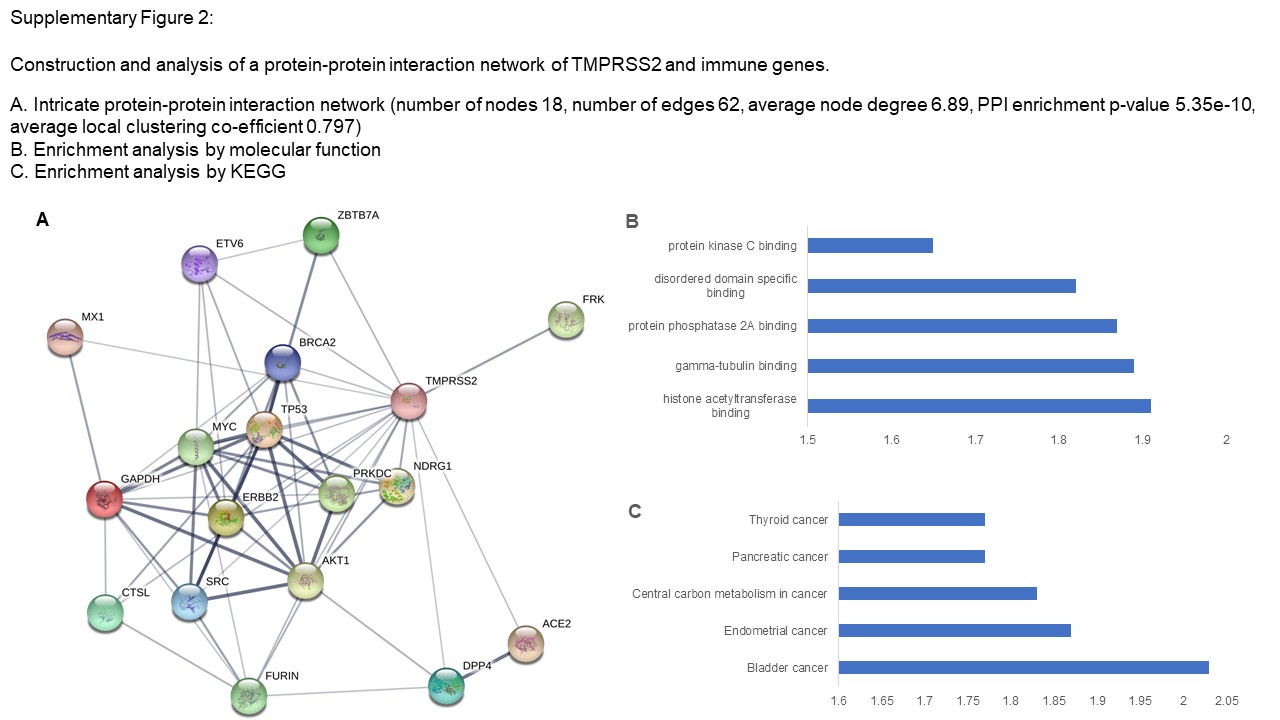

Supplement: Supplementary file 1 [file Data_Sheet_1.ZIP › Supplementary Figure 2.JPG]

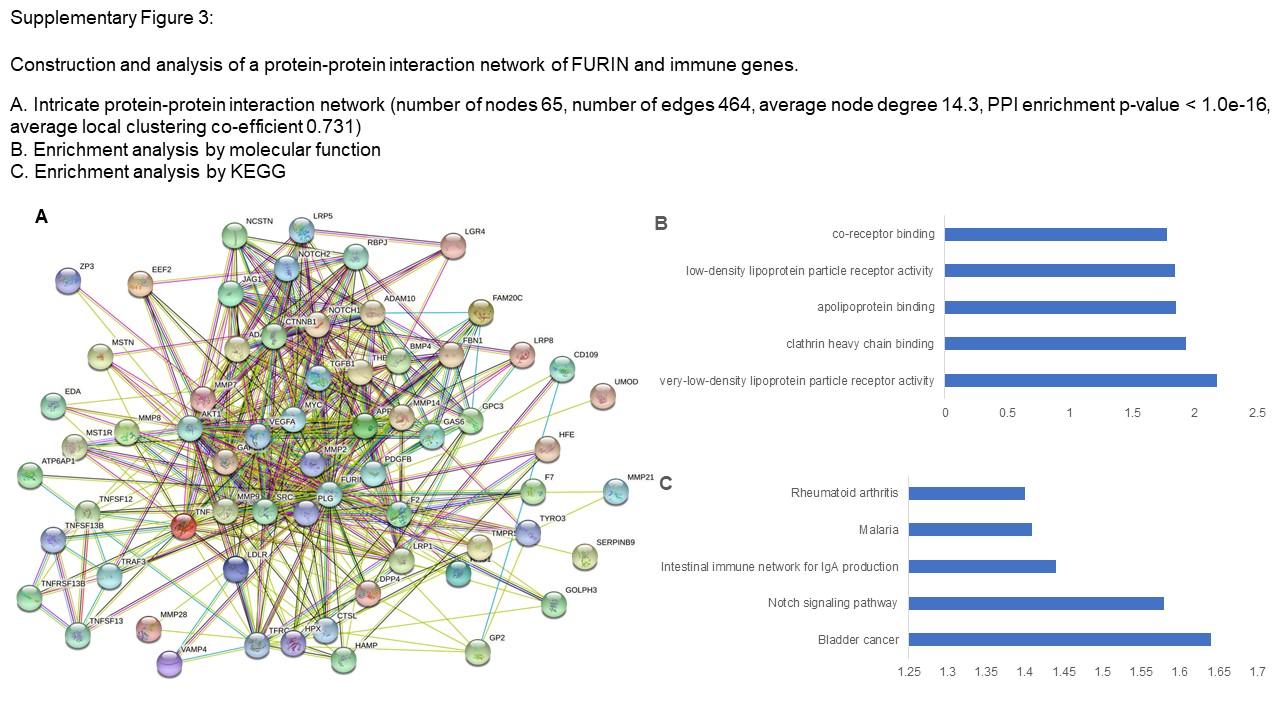

Supplement: Supplementary file 1 [file Data_Sheet_1.ZIP › Supplementary Figure 3.JPG]
